# Quality-of-life measures in pharmacogenomic studies: a systematic review

**DOI:** 10.1007/s11136-025-04030-w

**Published:** 2025-07-18

**Authors:** Shen Chi Ng, Nathan He, Patrick Fu, Senuri Mahavithana, Seoyoung Jang, Dina Abushanab, Zanfina Ademi

**Affiliations:** 1 Health Economics and Policy Evaluation Research (HEPER) Group, Centre for Medicine Use and Safety, Faculty of Pharmacy and Pharmaceutical Sciences, Monash University, 381 Royal Parade, Parkville, VIC 3052 Australia; 2 Department of Pharmacy, Hamad Medical Corporation, Doha, Qatar; 3 School of Public Health and Preventive Medicine, Monash University, Melbourne, Australia

**Keywords:** Quality of life, Pharmacogenomic, Generic, Specific, Systematic review

## Abstract

**Background:**

There are various quality of life (QoL) tools utilised in pharmacogenomic studies, but it remains unclear which tools are most frequently used. Our aim was to identify the types of QoL measures currently used in pharmacogenomic studies and address the existing evidence gap.

**Methods:**

A systematic review screened PubMed, Cochrane Library, Ovid, and Embase from inception through April 30, 2024. The search terms were “Pharmacogenetics” AND (“quality of life” OR “PROMS” OR “PREMS” OR “health related quality of life” OR “'EuroQol” OR “utility” OR “generic” OR “time trade off” OR “standard gamble” OR “SF-6D” OR “EQ-5D”). Our inclusion criteria were randomised clinical trials, cohort studies and cross-sectional studies that utilised generic and/or disease-specific QoL measures related to pharmacogenomics.

**Results:**

Twelve studies met the inclusion criteria, from which we identified the following tools: EORTC QLQ-C30 (n = 3), SF-36 (n = 3), SF-12 (n = 1), WHOQOL-BREF (n = 1), Q-LES-Q-SF (n = 1), FACT-Melanoma (n = 1), QLQ-C30 + QLQ BN20 (n = 1), QLQ-C30 + QLQ-CIPN20 (n = 1). The SF-36, SF-12, WHOQOL-BREF, and Q-LES-Q-SF are generic QoL questionnaires, while FACT-Melanoma, QLQ BN20, and QLQ-CIPN20 are disease specific. The EORTC QLQ-C20, although generic, is tailored for cancer patients. None of the included studies justified their choice of quality-of-life tool, nor was there consistency in how scores were reported in terms of overall and domain-specific outcomes.

**Conclusions:**

Pharmacogenomic studies employed diverse QoL instruments, hindering consistent and reliable reporting. Future studies should justify QoL tool selection and report both overall and domain-specific outcomes consistently to enable valid comparisons and inform decision-making.

**Supplementary Information:**

The online version contains supplementary material available at 10.1007/s11136-025-04030-w.

## Introduction

Pharmacogenomics is a rapidly evolving field that combines pharmacology and genomics to understand how an individual’s genetic makeup influences their response to medications [1]. By tailoring drug selection and dosage based on a patient's genetic profile, pharmacogenomics holds the potential to optimise treatment efficacy and minimise adverse drug reactions [2]. If pharmacogenomics can optimise an individual's health, it will positively affect their quality of life (QoL). Therefore, integrating quality-of-life measures in pharmacogenomic studies is crucial, as it ensures personalised treatment that enhances overall well-being. The World Health Organisation defines QoL as an individual’s perceived position within the context of their culture value systems, personal ambitions, expectations, standards, and concerns [3]. A variety of QoL instruments have been used across health-economic research [4] yet their application in pharmacogenomics remains poorly characterised. For example, Arnold et al. [4] compared indirect preference-based instruments (e.g., European quality of life five dimensions (EQ-5D), short form six dimensions (SF-6D), and health utilities index (HUI)) with direct elicitation techniques (standard gamble, time trade-off) and found that the former systematically produce lower utility scores. In the pharmacogenomic context, O’Shea et al. [5] evaluated genotype-guided therapy in patients with multimorbidity and polypharmacy, demonstrating reductions in hospitalisations, costs, and adverse drug events. Although they list QoL among their outcomes, they neither report specific QoL scores nor name the instruments used. Consequently, it remains unclear which QoL tools are most commonly applied in pharmacogenomic studies and how their results are presented. We conducted a systematic review by analysing pharmacogenomic studies that assessed QoL using data from selected databases. Our study aimed to identify the QoL tools currently in use, and to determine whether these studies employ generic tools, disease-specific tools, or a combination of both. Additionally, we investigated whether the studies provided justification for their choice of QoL tools and how they reported their scoring system (i.e. domain-specific vs. overall scores) which is central for ensuring the appropriateness, validity, reliability and comparability of the findings.

## Methods

The Preferred Reporting Items for Systematic reviews and Meta-Analyses search extension (PRISMA-S) guidelines was considered for this systematic review [6]. The PRISMA-S checklist is provided in Appendix 1.

We conducted a systematic review and used a search strategy through Cochrane, Embase, Ovid and PubMed. The literature search was conducted from inception until April 30, 2024. We assigned two members to search through Cochrane, and one member for each of the other databases. We entered the same search terms, “Pharmacogenetics” AND (“quality of life” OR “PROMS” OR “PREMS” OR “health related quality of life” OR “'EuroQol” OR “utility” OR “generic” OR “time trade off” OR “standard gamble” OR “SF-6D” OR “EQ-5D”) into the databases. However, on PubMed, using the term “utility” produced too many results, so we entered “health utility” instead to narrow down the results. The keywords were selected by considering the titles and abstracts of full publications relevant to our study, areas our study covers, and names of QoL tools. The inclusion of terms like 'time trade off' and ‘standard gamble’ was intentional, as these are direct utility-elicitation methods used to measure the health-state utility, which are essential for calculating Quality-Adjusted Life Years (QALYs) and are often reported alongside QoL data. This strategy ensured a comprehensive capture of studies while focusing on those that report QoL measures, which are important for understanding the impact of pharmacogenomic interventions on patient QoL.

Our inclusion criteria consisted of randomised clinical trials, cohort studies and cross-sectional studies. Both generic and disease-specific QoL measurements related to pharmacogenomics were included. Our exclusion criteria consisted of non-English and non-human studies, abstracts in the publications that mentioned QoL with no relation to pharmacogenomics, mention of pharmacogenomics but no specification to the QoL tool used, and studies without full text. Studies that mentioned the EQ-5D questionnaires without the specific version of the tool, or studies that utilise EuroQol VAS scale alone were excluded as well. Detailed reasons for the exclusion of studies are provided in Appendix 2. After obtaining our initial search results, each team member performed title and abstract screening on their assigned database. During this process, we evaluated every record against our predefined inclusion and exclusion criteria, documenting reasons for including or excluding each article. We also identified and removed duplicates across databases. For each database, we recorded the total number of hits retrieved, the number of duplicates removed, and the counts of articles included and excluded.

**Fig. 1:**
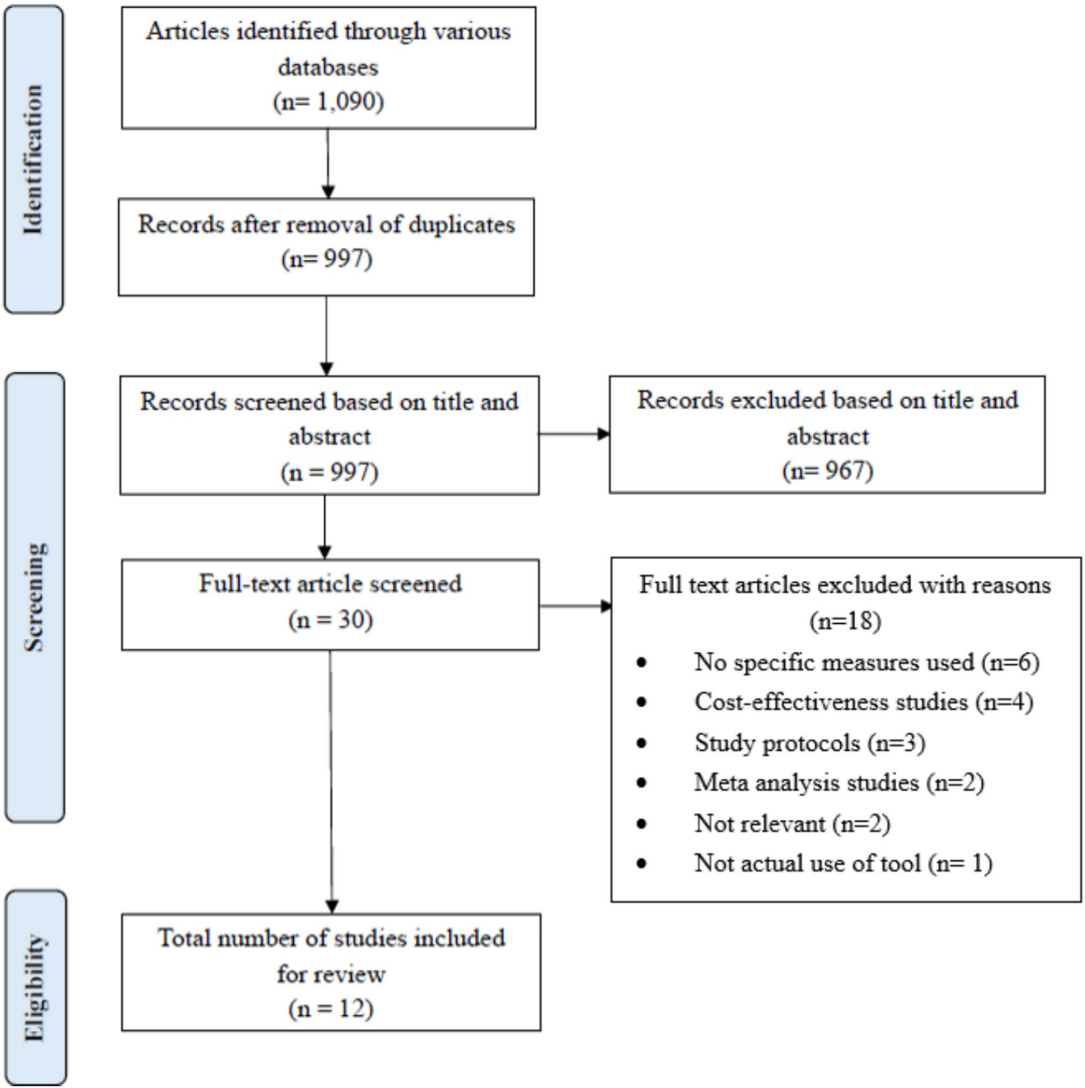
PRISMA flow diagram. The flow of information through the various phases of this systematic review, including the number of records identified, excluded, and included

Upon completing title and abstract screening, we compiled the studies deemed eligible into a master Excel spreadsheet to facilitate full‐text review. ach article underwent two independent screenings: first by the reviewer who performed its initial title/abstract assessment, and subsequently by a second team member to verify data accuracy. We extracted the following information for each study: the type and name of the QoL instrument, the authors’ rationale for tool selection, whether the measure was generic or disease-specific, and whether domain-specific or overall utility scores were reported. We also recorded which team member screened each article (and when), along with study authorship, publication year, country, language, clinical condition, participant demographics (age and recruitment source), study design, a brief description of the intervention or exposure, and funding source. We applied our predefined inclusion and exclusion criteria at this stage, recording reasons for exclusion where applicable Once each article had been screened twice, we obtained our final set of included papers and confirmed with the third reviewers (ZA and DA). The completed PRISMA flow diagram was used to summarise our search strategy and screening across all the databases (Fig. 1). Although we initially planned to include risk of bias assessment in the PROSPERO protocol, we did not pursue it, given the exploratory aim of this review, to identify and characterise the QoL instruments employed in pharmacogenomic studies rather than to evaluate intervention effectiveness. This study has been registered in PROSPERO with the following number CRD42024542177.

## Results

### Search results

Our search yielded a total of 1090 titles from the databases; 93 duplicates were removed and leaving us with 997 unique titles. After title and abstract screening of these articles, 967 articles did not meet our inclusion criteria and hence were excluded. We were left with 30 articles to commence our full text screening on. Ultimately, 18 of these articles were excluded. Of which, five of the articles explored topics that were not relevant to our study, seven of them utilised study designs that were not a part of our inclusion criteria, one of them did not have an accessible full text, four of the articles did not mention the specific QoL tool that was utilised, and one study reported cost effectiveness and QALY, but not QoL scores. Consequently, twelve published articles remained and were included in the final review.

### Study design

**Fig. 2:**
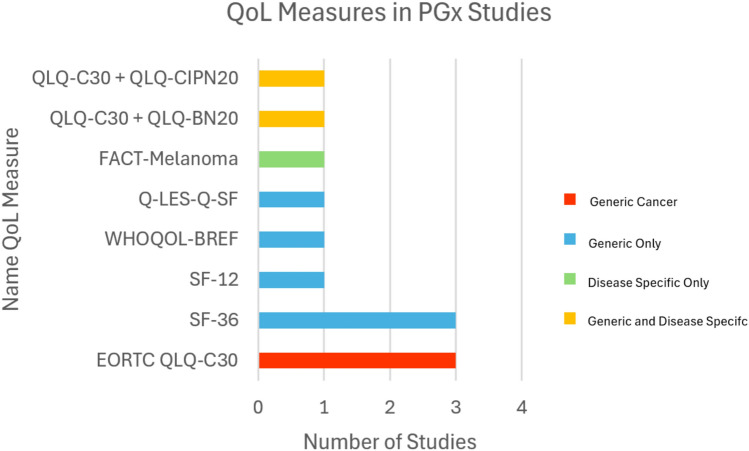
Summary of the different types of QoL measures identified in our included pharmacogenomic studies

Tables 1 and 2 provide a description of the pharmacogenomics studies which includes author, year of publication, country, the study population, study design, sample size, type of pharmacogenetic test and gene tested, and the drug tested in the study. Furthermore, Table 1 describes the characteristics of the QoL measures used in each included study, and whether justification was provided for the selected QoL tools. Figure 2 summarises the different types of QoL measures identified in the included studies. Table 3 lists the QoL scores obtained from each of the studies’ selected QoL tools.

**Table 1:** Characteristics of the QoL measures used in pharmacogenomic studies

Author Year of Publication	Type of Quality of Life Measure	Name of Quality of Life Measure	Description of Quality of Life Measure	Justification for Selecting the Quality of Life Measure	Source of Population	Study Funding Source
Lebbé et al. (2020)	Disease-specific	FACT-Melanoma	FACT- Melanoma is a validated QoL measurement designed specifically used in patients with melanoma, which incorporates 4 domains: physical well-being, social/family well-being, emotional well-being, and functional well-being, and melanoma-specific issues and symptoms	No	Patients with untreated NRAS-mutated melanoma ranged in ages of 21–83, multicentre	Merck Healthcare KGaA
Riccetti et al. (2022)	Generic cancer	EORTC QLQ-C30	EORTC QLQ-C30 scale, a 30-item instrument that measures QoL in cancer patients in three main domains: global health status, functional status, and cancer-related symptom status	No	Patients from University Hospitals in Mainz, Frankfurt, Leipzig, Freiburg and Homburg, and the Catholic Hospital in Mainz, age ≥ 18 y/o, have lung cancer-related admission to hospital & being mentally and verbally able to take part in a telephone interview in German	“Cancer Prevention, Early Detection, and Outcomes” of the German Cancer Consortium (DKTK)
Oberkampf et al. (2023)	Generic cancer, disease specific	EORTC QLQ-C30, QLQ BN20	EORTC QLQ-BN20 was developed and validated to evaluate the effects of the tumour and its treatment on symptoms, functions and HRQoL of brain tumour patients, both in clinical trials and clinical practice	No	Patient with HER2 positive breast cancer and LM diagnosed by CSF cytology ± clinical symptoms of LM and LM evidence on MRI. Patients with symptomatic brain metastases included if surgery ± radiotherapy performed at least 3 weeks before inclusion	Roche group
Hajj et al. (2022)	Generic cancer	EORTC QLQ-C30	As above	No	Women aged ≥ 18, with a primary diagnosis of breast cancer, and admitted to the outpatient oncology unit at Hôtel-Dieu de France for intravenous chemotherapy every 21 days (random cycle out of a maximum of 10 cycles)	"Conseil de la recherche" of the Saint-Joseph University
Ciruelos et al. (2019)	Generic cancer, disease specific	EORTC QLQ‐C30, QLQ‐CIPN20	EORTC QLQ-C30 – as above QLQ-CIPN20 was developed to assess the impact of chemotherapy-induced peripheral neuropathy on the QoL of cancer patients, which consists of 20 items divided into three subscales that encompass specific sensory, motor, and autonomic symptoms associated with chemotherapy-induced peripheral neuropathy	No	Women aged ≥ 18 years with histologically or cytologically confirmed and measurable (RECIST criteria) stage IV breast cancer, an Eastern Cooperative Oncology Group performance status of 0–1, metastatic HER2‐negative breast cancer with no prior chemotherapy for metastatic disease, and adequate organ function who were able and willing to provide two plasma samples for pharmacogenetic analysis	Oncosur, Celgene Corporation
Hajj et al. (2017)	Generic cancer	EORTC-QLQ-C30 v1.0	EORTC QLQ-C30 v1.0 is the second generation of EORTC QLQ-C30, that incorporates 5 functional scale (physical role, cognitive, emotional, and social), 3 symptom scales (fatigue, pain and nausea and vomiting), a global health status/ QoL scale, and additional symptoms commonly reported by cancer patients, and perceived financial impact of the disease	Lacked a specific rationale for why EORTC-QLQ-C30 measure was the most appropriate choice	Patients > 18 years old, diagnosed as suffering from malignant diseases, and had received scheduled morphine treatment corresponding to step III at the analgesic ladder of the WHO	“Conseil de la Recherche” of the Saint-Joseph University and the “National Council for Scientific Research in Lebanon (CNRS-L)”
Brennan et al. (2015)	Generic	Q-LES-Q-SF	Q-LES-Q-SF is a type of generic quality-of-life measure, consists of 16 items that cover various aspects of life focusing on enjoyment and satisfaction	No	Clinicians with valid national provider identifier number & able to complete online questionnaires, who ordered Genecept Assay for the respective Psychiatric patient > 18 y/o, had ability to complete electronic informed consent	Genomind, Inc, King of Prussia, Pennsylvania
Bohlen et al. (2022)	Generic	SF-36	SF-36 is a generic quality-of-life measure that widely used 36-item instruments that covers 8 domains to assess overall health status, including physical, mental, and social functioning with each domain scored on a 0–100 scale	No	Participants ≥ 18 with MDD or DDNOS per DSM-V were recruited from one health system located in four Midwestern states in the US (SD, MN, IA, NE)	Avera Institute for Human Genetics
Agulló et al. (2023)	Generic	SF-12	SF-12 is a condensed version of SF-36, consisting of 12 items derived from the SF-6 covering 8 health domains to assess overall health status	No	Adults aged ≥ 18 years with CNCP who required opioid analgesic treatment	Instituto de Salud Carlos III
Martin et al. (2019)	Generic	SF-36	As above	No	Participants ≥ 18 years of age, suffering from peripheral NP excluding central or diabetic origin and relieved by ketamine. They had to be registered to the French Healthcare system	Regional Hospital and Fondation de France
Isaza et al. (2023)	Generic	WHOQOL-BREF	WHOQOL-BREF estimates the QoL in four areas: health and physical health, psychological health, social relationships, and environment	Yes	Patient age between 17 and 40 years, a diagnosis of opioid dependence based on the DSM IV criteria, acceptance of the study requirements and provision of informed consent, and no MMT within the previous six months	Universidad Tecnológica de Pereira
Schricker et al. (2021)	Generic	SF-36	As above	No	Participants aged > 18 years who agreed to genetic testing and to participate in the study	Robert Bosch Stiftung

FACT: Functional Assessment of Cancer Therapy, EORTC QLQ: European Organization for the Research and Treatment of Cancer Quality of Life Questionnaire, HRQoL: health-related quality of life, QoL: quality of life, SF-36: 36-Item Short-Form Health Survey, WHOQOL-BREF: World Health Organization Quality of Life scale, WHO: World Health Organization, MDD: major depressive disorder, DDNOS: depressive disorder not otherwise specified, DSM-V: Diagnostic and Statistical Manual of Mental Disorders—5th Edition, SDDGI: significant drug-drug-gene interaction, CNCP: chronic non-cancer pain, NP = neuropathic pain, DSM IV: Diagnostic and Statistical Manual of Mental Disorders—4th Edition, MMT: methadone maintenance treatment, SD: standard deviation

**Table 2:** Description of the pharmacogenomic studies that utilised QoL measures, including the study design and specific pharmacogenomic interventions employed

Author Year of publication Country	Population (Medical Condition)	Study design	Sample Size	Type of PGx Test Type of gene tested	Study description Drug tested
Lebbé et al. (2020), Australia, New Zealand, Europe, North America	Untreated NRAS-mutated melanoma	RCT	194	Centralized N-RAS mutations status tested using Sanger technique	Dacarbazine (n = 64) or pimasertib (n = 130) on the first day of their 21-day cycle
Riccetti et al. (2022), Germany	Lung cancer	Observational cross-sectional study	665	RTK alterations were tested. No technique specified	Group A had proven RTK alterations, TKI therapy at any time during therapy and stage IV lung cancer at diagnosis (n = 49). Group B had non-TKI therapy and stage IV lung cancer (n = 121). Group C had non-TKI therapy and stage I-III lung cancer (n = 495)
Oberkampf et al. (2023), France	HER2-positive breast cancer with leptomeningeal metastasis	Observational prospective cohort study	19	FCGR3A and FCGR2A genotypes tested with QuickGene-610L Fujifilm extractor	Weekly administration of IT trastuzumab
Hajj et al. (2022), Lebanon	Breast cancer	Observational cross-sectional study	67	COMT, DRD2, OPRM1, CLOCK, PER2, CRY2, ABCB1 were tested using Lightcycler®	Patients receiving chemotherapy complete a self-reported questionnaire that included several validated scales to evaluate fatigue, sleep, anxiety, depression, and pain
Ciruelos et al. (2019), Spain	Breast cancer	RCT	60	CYP3A4*20, CYP3A4*22, CYP2C8*3, ABCB1, EPHA5, EPHA6 and EPHA8 were tested using KASPar Technology on a Sequence Detection System ABI PRISM 7900HT	4-week cycles of PACL80/w (n = 14), NAB100/w (n = 16) or NAB15/w (n = 14) on day 1, 8, and 15, or NAB150/2w (n = 16) on day 1 and 15
Hajj et al. (2017), Lebanon	Malignant disease	Observational prospective cohort study	89	OPRM1, ABCB1, COMT were tested using Lightcycle® 2.0	Patients were treated by morphine for different types of cancer including gastrointestinal tract (n = 20), breast (n = 16), lung (n = 15), hematologic (n = 9), urogenital (n = 6), gynecologic (n = 6), prostate (n = 4), pancreas (n = 4), head and neck (n = 2), sarcoma (n = 2) and others (n = 5)
Brennan et al. (2015), United States	Psychiatric diagnosis, primarily depression and anxiety	Observational cross-sectional study		CYP2D6, CYP2C19, CYP3A4, 5HT2C, DRD2, CACNA1C, ANK3, COMT and MTHFR were tested using TaqMan Genecept Assay test	Patients (n = 625) complete 4 questionnaires at baseline, 1 month and 3 months to measure depression, anxiety, QoL, side effects and demographic questionnaire Clinicians (n = 42) complete CGI-S scale for disease severity & treatment plan after review of genetic test results within 1 week of receiving genetic result and 3 months later
Bohlen et al. (2022), United States	MDD, DDNOS	Observational prospective cohort study	175	CYP1A2, CYP2B6, CYP2C9, CYP2C19, CYP2D6 + CYP2D6 copy number: Exon 9, CYP3A4, OPRM1, HTR2A, and COMT were tested using PCR on the Applied Biosystems ViiA 7 Real-Time PCR System. Genotype calls made using TaqMan® Genotyper Software v1.31 by Life Technologies. 5-HTTLPR and rs25531 were tested using RFLP with MspI	Pharmacogenetic testing outcomes of participants managed in primary care (PC) (n = 95) and psychiatric care (PSY) (n = 80)
Agulló et al. (2023), Spain	CNCP	RCT	50	OPRM1, COMT and CYP2D6 were tested using the real-time PCR rotor gene Q system with specific TaqMan MGB® probes	PGx-guided arms (n = 28) vs usual prescribing (n = 22) to analyse clinical changes after 3 months of opioid treatment
Martin et al. (2019), France	Peripheral neuropathic pain excluding central or diabetic origin	RCT	60	CYP2D6*6 was tested using a long PCR method for whole-gene amplification followed by subsequent nested PCR and restriction enzyme analysis CYP2D6*3, CYP2D6*4, CYP3A4*1B, CYP3A4*22, CYP3A5*3, ABCB1, NR1I2 were tested by Taq Man® Drug Metabolism Genotyping Assays	Oral dextromethorphan (n = 20), memantine (n = 20) or placebo (n = 20) after their ketamine infusion for 12 weeks
Isaza et al. (2023), Colombia	Opioid (heroin) dependence	RCT	72	ABCB1, CYP2B6, and OPRM1 were tested using the mini-sequencing method. Data were analyzed based on the peaks’ colors and the fragments’ sizes using Genemapper V3.2 software	Conventional treatment (n = 34) vs genetic markers were used to calculate the methadone dose (n = 38) to assess retention rate, heroin usage and quality of life
Schricker et al. (2021), Germany	Risk genes for malignant, cardiovascular, coagulation, storage diseases and pharmacogenetics	Observational prospective cohort study	244	Genetic panel which includes 7 “modules” on risk variants BRCA1, SPINK1, MUTYH, PALB2, LZTR1, BRCA2, MLH1, NBN, TP53, SDHAF2, MYBPC3, MFAP5, SCN5A, F5, F2, FBN1, VWF, compound heterozygote, LDLR	Patients provided demographic and clinical data, family history, anamnesis and diagnoses at baseline prior initiation of genetic testing, and were assessed by 4 questionnaires at baseline and in follow-up (at least 3 months after return of genetic test results)

MDD: major depressive disorder; DDNOS: depressive disorder not otherwise specified, CNCP: chronic non-cancer pain, PFS: progression-free survival, RFLP: restriction length fragment polymorphism

**Table 3:** Results of the studies’ selected QoL tool, including whether they reported overall score or specific domain score

Author Year of Publication	Result of Quality of Life Assessment
Lebbé et al. (2020)	FACT–General total mean (SD) scores for pimasertib vs dacarbazine were: Baseline: 80 (16) vs 75 (15) End of treatment: 74 (17) vs 70 (15)
Riccetti et al. (2022)	Mean (SD) global QoL for: Grp A (confirmed RTK alteration & TKI therapy, stage IV) = 57.1 (21.4) Grp B (confirmed no RTK alteration & TKI therapy, stage IV/ metastases) = 68.8 (22.8) Grp C (confirmed no RTK alteration & TKI therapy, stage I-III & no metastases) = 57.5 (24.6)
Oberkampf et al. (2023)	The median (min–max) score for QoL improved/steady after 8 weeks of treatment: Week 1: 61 (42–74) Week 5: 64 (37–73) Week 9: 58 (53–67)
Hajj et al. (2022)	Mean (SD) fatigue score of 42.12 (32.10)
Ciruelos et al. (2019)	Patients in the NAB150/w group exhibited the greatest increases in each scale of the EORTC QLQ‐CIPN20, and the difference with patients in the PACL80/w group was only significant for the sensory scale (estimated treatment difference [ETD], 19.0; 95% confidence interval [CI], 1.1–37.0; p = .039
Hajj et al. (2017)	The Mean (SD) score of global health status (GQOL) is 33.74 (26.67) versus 51.24 (29.57)
Brennan et al. (2015)	Mean Q-LES-Q-SF score: -Baseline: 44.7 -By month 1: 48.5 -By month 3: 50.0
Bohlen et al. (2022)	Quality of life (SF-36): Physical Quality of life score -Change over time Beta: 0.07/p-value: 0.6109 -Randomisation Beta: 0.55/p-value: 0.7268 Mental Quality of life score -Change over time Beta: 2.32/p-value < 0.0001 -Randomisation Beta: 0.55/p-value: 0.5457
Agulló et al. (2023)	Mean (SD) SF-12 physical component score for PGx-guided group improved from 27 (7) to 36 (80), usual care group improved from 28 (7) to 29 (9) Mean (SD) SF-12 mental component score Baseline for PGx-guided group = 43 (14) Baseline for usual care group = 41 (12) Final for PGx-guided group = 43 (7) Final for usual care group = 43 (12)
Martin et al. (2019)	Mean (SD) SF-36 score at month 2: Dextromethorphan = 50.06 (19.08) Memantine = 53.06 (23.08) Placebo = 34.06 (20.23) Mean (SD) SF-36 score at month 3: Dextromethorphan = 32.35 (18.88) Memantine = 48.44 (14.80) Placebo = 37.14 (14.64) Mean (SD) SF-36 score between 3 days after ketamine infusion and month 3: Dextromethorphan = − 25.75 (49.08) Memantine = 46.97 (111.51) Placebo = − 57.14 (46.00)
Isaza et al. (2023)	WHOQOL-BREF was not used when patients first started methadone maintenance treatment. The eighth week of treatment indicated that their quality of life had improved significantly since beginning the program. No explicit mean, median, or standard deviation values for the WHOQOL-BREF scores were provided
Schricker et al. (2021)	The SF36 mental and physical subscales, quality of life indicators, showed no significant differences between baseline and follow-up for the entire cohort. However, overall positive changes were observed in health-related behaviour, physical activity, and nutrition. The quantitative data was presented in Fig. 2

FACT: Functional Assessment of Cancer Therapy, EORTC QLQ: European Organization for the Research and Treatment of Cancer Quality of Life Questionnaire, HRQoL: health-related quality of life, QoL: quality of life, SF-36: 36-Item Short-Form Health Survey, WHOQOL-BREF: World Health Organization Quality of Life scale

### Study characteristics

A total of twelve studies investigated pharmacogenomics and QoL within patient populations. These comprised five randomised controlled trials [7–11], four observational prospective cohort studies [12–15] and three observational cross-sectional studies [16–18] (See Tables 1 and 2).

Among these studies, six studies were performed in Europe, specifically in Spain [7, 9], France [11, 14], and Germany [15, 18]. Of the studies included, two were conducted in the Middle East, specifically in Lebanon [13, 17], and two were conducted in the United States [12, 16]. Additionally, one study took place in Colombia [8], and another included multi sites; Australia, New Zealand, Europe, and North America [10]. In terms of publication dates, four studies were published in the 2010s (2015, 2017, 2019), and eight studies were published in the 2020s (2020–2023) (See Tables 1 and 2).

Six studies investigated cancer-related diseases, with three of these addressing breast cancer [7, 14, 17] and one each examining neuroblastoma ras viral oncogene homolog (NRAS)-mutated melanoma [10], lung cancer [18], and multiple malignant diseases (See details in Tables 1 and 2). Two studies explored mental health conditions. One study investigated psychiatric disorders that primarily focused on depression and anxiety [16], whilst the other on depressive disorders [12]. Three studies investigated pain-related conditions. Two of these cantered on chronic non-cancer pain [9] and peripheral neuropathic pain [11], while the third investigated heroin dependence [8]. Additionally, one study examined the testing of over 100 risk genes for malignant, cardiovascular, coagulation, storage diseases, and pharmacogenetics [15] (see details in Tables 1 and 2). Along this type of PGx testing performed in each of the studies was recorded (see details in Tables 1 and 2).

### Cancer related studies

Out of the six cancer-related studies, five studies had utilised the EORTC-QLQ-C30 tool, which is a generic cancer QoL measurement. In the study by Hajj et al., version 1 of the EORTC-QLQ-C30 had been used specifically [13]. Among these five studies, two of them had concurrently used a disease-specific questionnaire. The study by Oberkampf et al. utilised QLQ BN20 [14], while Ciruelos et al.’s study chose the QLQ-CIPN20 tool [7]. Only one of the cancer-related studies had used a disease-specific measurement alone, which was the FACT-Melanoma tool [10] (Table 2 and Fig. 2).

Out of the six cancer studies, five of them did not provide any justification for their selection of QoL tools [7, 10, 14, 17, 18] (Table 1). The study by Ciruelos et al. [7] and the 2022 study by Hajj et al. [17] reported a domain specific score, while the rest reported the overall QoL score. The results of the cancer studies and their selected QoL tools, including whether they reported the overall QoL score, or a specific domain score was also reported (See Table 3).

### Mental health related studies

Both mental health studies used generic measurements, which were Q-LES-Q-SF and SF-36 (Table 2 and Fig. 2). Neither of studies provided any justification for the selection of their QoL tools [12, 16] (Table 1). The study by Brennan et al. reported the mean Q-LES-Q-SF score [16], whereas the study by Bohlen et al. derived the physical and mental QoL subscale from SF-36 into a linear regression model [12] (Table 2). Mental health related studies did not state the overall health related QoL scores (See Table 3).

### Pain related studies

The three pain-related studies had utilised generic tools, the first study employed SF-12 [9], the second study used SF-36 [11], and the third study used WHOQOL-BREF [8] (Table 2 and Fig. 2). Isaza et al. (i.e. WHOQOL-BREF) [8] used QoL as an outcome measure in the context of methadone treatment, a common intervention for managing both addiction and associated pain symptoms. The study by Agulló et al. as well as the study by Martin et al. did not justify their QoL tool selection [9, 11]. The study by Isaza et al. justified their QoL tool choice [8] (Table 1). The study by Agulló et al. reported the mean score for the physical component of the selected tool [9]. The study by Martin et al. reported the overall mean [11]. Meanwhile, the study by Isaza et al. simply stated whether there was change in QoL over a period of eight weeks and did not report any baseline QoL data [8]. (Table 3).

### Other studies

The final study looked at several panels and selected the SF-36 (Table 2 and Fig. 2). However, this study did not provide justification for selecting SF-36 [15] (Table 1). This study reported whether there were changes in QoL between baseline and follow-up, specifically regarding the SF-36 mental and physical subscales. These subscales, which are key indicators of QoL, showed no significant differences for the entire cohort. Also, the study did not report the overall QoL scores [15] (Table 3).

### Psychometric properties of the QoL in the included studies

Validity of the QoL instruments was reported in four studies: Hajj et al. [13], Hajj et al. [17], Agulló et al. [9], and Isaza et al. [8]. Reliability was only mentioned by Isaza et al. [8]. Minimal clinically important difference (MCID) reporting was also limited, with only Martin et al. [11] reported that a sample size of 20 patients allowed to detect a true a minimal difference greater than 1.4 points on the SF-36.

## Discussion

This systematic review aimed to investigate the types of QoL measures used in pharmacogenomic studies. We found that a variety of questionnaires, particularly disease-specific measures, were utilised, which have not been extensively explored in previous research. However, this diversity also reflects a lack of consistency in QoL assessment and specifically reporting practices (i.e. domain-specific vs. overall scores) across studies.

A significant finding from this systematic review was that most of the studies reported domain-specific outcomes rather than overall QoL utility scores [9–11, 18]. This is particularly concerning, as domain scores cannot be directly used in health economic evaluations. These analyses rely on utility values (i.e. overall scores) for the prioritisation of healthcare resources. The absence of overall utility scores limits the ability to compare interventions or prioritise treatments based on their impact on patients’ QoL.

Moreover, most studies did not fully report how their QoL instruments performed in terms of reliability and validity. Only Isaza et al. [8] provided both reliability and validity evidence. Three others (Hajj et al. [13]; Hajj et al. [17]; Agulló et al. [9]) reported validity alone, and only Martin et al. [11] noted the minimally clinically important difference. This lack of psychometric detail limits our ability to interpret and generalise QoL findings. Consistent reporting of reliability, validity, and responsiveness is therefore essential for robust QoL assessment in pharmacogenomic research.

The most common generic QoL measure used in the studies was the SF-36, which was employed by Bohlen et al. [12]., Martin et al. [11]., and Schricker et al. [15]. This is a widely used 36-item instrument that covers eight domains to assess overall health status, including physical, mental, and social functioning [19]. The SF-36 is applicable across a wide range of diseases and conditions, making it suitable for use in various patient groups. Another generic QoL measure that was utilised was SF-12 in the study by Agulló et al. [9], a condensed version of the SF-36, designed to provide a concise measure of health-related QoL [20]. The World Health Organisation developed WHOQOL-BREF [3], another generic QoL tool, which was used in the study conducted by Isaza et al. [8]. It is designed to provide a comprehensive assessment of an individual's QoL across 4 domains: physical health, psychological health, social relationships, and environment. The Q-LES-Q-SF, a generic QoL measure used by Brennan et al. [16]., is a short form of the original Q-LES-Q, consists of 16 items that cover various aspects of life focusing on enjoyment and satisfaction, derived from the original 93-item form [21].

For pharmacogenomic studies in cancer patients, the EORTC-QLQ-C30 questionnaire was frequently used. This is a generic, 30-item instrument that measures QoL in all cancer patients across key health domains such as physical functioning, emotional functioning, fatigue and pain [21]. Both studies, Riccetti et al. [18] and Hajj et al. [17] used this tool in their respective pharmacogenomic studies.

Only one of the studies employed a disease-specific measurement. In this study, Lebbé et al. [10] used the FACT-Melanoma tool to assess the QoL in patients with melanoma. FACT-Melanoma is one of the two validated QoL tools for use in melanoma patients, and it incorporates four main domains: physical well-being, social/family well-being, emotional well-being, and functional well-being [22]. The melanoma-specific subscale focuses on issues and symptoms particularly relevant to melanoma patients, such as skin symptoms, side effects of treatment, and other melanoma-specific QoL concerns.

Some pharmacogenomic studies used both generic and disease-specific QoL measures. For instance, studies conducted by Oberkampf et al. [14] and Ciruelos et al. [7] used EORTC QLQ-C30 as a generic QoL tool for cancer. For the disease-specific measure, Oberkampf et al. [14] and Ciruelos et al. [7] used QLQ-BN20 and QLQ‐CIPN20, respectively. The QLQ-BN20 was designed to measure the QoL in patients with brain tumours, including the specific symptoms and concerns associated with this condition [23]. The QLQ-CIPN20 was developed to assess the impact of chemotherapy-induced peripheral neuropathy on the QoL of cancer patients. The questionnaire consists of 20 items divided into three subscales that encompass specific sensory, motor, and autonomic symptoms associated with chemotherapy-induced peripheral neuropathy [24].

While most studies included in this review did not provide explicit justifications for their choice of QoL instruments, Isaza et al. [8] highlighted the reliability of the WHOQOL-BREF in assessing QoL among heroin-dependent patients receiving methadone. However, even in this case, the justification was relatively broad and lacked specificity regarding context and population relevance. Similarly, Hajj et al. [13] acknowledged the EORTC-QLQ-C30 as a widely recognised and validated assessment tool, their justification for its use in assessing QoL in palliative cancer patients receiving morphine was insufficient. They did not provide a clear rationale for why this generic QoL measure was the most suitable choice, especially considering the availability of disease-specific modules that might have been more appropriate for this patient population. Furthermore, the study did not discuss the sensitivity of EORTC-QLQ-C30 to changes in QoL within this context or compare it to other potentially more suitable measures. In their discussion, the authors themselves recognised that disease-specific modules could be more fitting, highlighting a gap in their rationale for selecting the EORTC-QLQ-C30 for their study. The absence of well-articulated rationales across studies limits the ability to evaluate whether the selected instruments were appropriate for capturing outcomes meaningful to patients. Clear justification for QoL instrument selection is essential for advancing methodological rigour and ensuring that tools are suitable for the intended population and research objectives.

### Strength and limitations

Our study has several key strengths. We employed a comprehensive search strategy across multiple databases to ensure thorough identification of relevant articles. Furthermore, the rigorous screening process, including extensive title and abstract screening, followed by full-text screening, and cross-checking by different reviewers, supports the reliability and reproducibility of our findings. However, these strengths should be considered alongside the limitations of our review. The exclusion of non-English studies means that our findings may not fully capture the nuances of QoL assessment in diverse cultural contexts. QoL is a complex and culturally influenced construct, and restricting our search to English-language publications may have introduced a bias. Additionally, the exclusion of grey literature may have resulted in overlooking relevant studies, particularly those with non-significant or negative findings. This could potentially lead to an overestimation of the consistency or strength of the observed patterns.

### Recommendations for future research and practice

Future studies should aim to report: (i) the rationale for instrument selection; (ii) the overall utility or summary scores (where applicable); (iii) domain-level results with descriptions of what is being measured; (iv) details regarding administration (e.g., timing, setting, and administrator); and (v) consistent use of standardised scoring formats. Improved transparency in these areas would enhance the interpretability, comparability, and overall quality of QoL data in pharmacogenomic and related research.

## Conclusion

Pharmacogenomic studies employed diverse QoL instruments, hindering consistent and reliable reporting. Future studies should justify QoL tool selection and report both overall and domain-specific outcomes consistently to enable valid comparisons and inform decision-making.

## Supplementary Information

Below is the link to the electronic supplementary material.

Supplementary file1 (DOCX 33 KB)

## Data Availability

All data analysed during this work are included in this published article. Any additional data are available on request from authors.
